# Davy on the Use of Narcotic Vapors

**Published:** 1847-06

**Authors:** 


2. *Sir Humphrey Davy on the Use of Narcotic Vapors in mitigating Pain.*—In the collected works of Sir Humphrey Davy, published in 1839, occur the following remarks on the effects of narcotic vapors in producing insensibility.

"In one instance, when I had headache from indigestion, it was immediately removed by the effects of a large dose of gas; though it afterwards returned, but with much less violence. In a second instance, a slighter degree of headache was wholly removed by two doses of gas.

"The power of the immediate operation of the gas, in removing intense physical pain, I had a very good opportunity of ascertaining.

"In cutting one of the unlucky teeth, called *dentes sapientæ*

I experienced an extensive inflammation of the gum, accompanied with great pain, which equally destroyed the power of repose and of consistent action.

"On the day when the inflammation was most troublesome, I breathed three large doses of nitrous oxide. The pain always diminished after the first four or five inspirations; the thrilling came on as usual, and uneasiness was swallowed up in pleasure.

"As nitrous oxide, in its extensive operation, appears capable of destroying physical pain, it may probably be used with advantage during surgical operations in which no great effusion of blood takes place."—*Bost. Med. and Surg. Jour.*

